# The Influence of Human Support on the Effectiveness of Digital Mental Health Promotion Interventions for the General Population

**DOI:** 10.3389/fpsyg.2021.716106

**Published:** 2021-08-19

**Authors:** Melanie Elise Renfrew, Darren Peter Morton, Jason Kyle Morton, Geraldine Przybylko

**Affiliations:** Lifestyle Medicine and Health Research Center, Avondale University College, Cooranbong, NSW, Australia

**Keywords:** digital mental health promotion, human support, human guidance, adherence, mental wellbeing, general population, interdisciplinary

## Abstract

Mental wellbeing amongst the general population is languishing—exacerbated by the Coronavirus Disease 2019 (COVID-19) pandemic. Digital mental health promotion interventions, that improve mental health literacy and encourage adoption of evidence-informed practical strategies are essential. However, attrition and non-adherence are problematic in digital interventions. Human support is often applied as an antidote; yet, there is a paucity of randomized trials that compare different human support conditions amongst general population cohorts. Limited trials generally indicate that human support has little influence on adherence or outcomes in DMHPIs. However, providing participants autonomy to self-select automated support options may enhance motivation and adherence.

## Introduction

As the world deals with the Coronavirus Disease 2019 (COVID-19) pandemic, a parallel crisis in mental wellbeing is emerging. Prior to the pandemic, depression was a leading cause of global disability (World Health Organization, 2019) and, in high income countries, depression and anxiety were the third and fourth highest reasons, respectively, for visiting a primary care physician (Finley et al., 2018). Furthermore, treatment coverage for mental health disorders was already extremely low, particularly in low income nations (World Health Organization, 2019). Alarmingly, extensive deterioration in mental wellbeing has been detected globally since the pandemic began (Ammar et al., 2020; Pera, 2020) across all sectors (e.g., general public, health workers, and those with pre-existing psychological conditions) (Vindegaard and Benros, 2020), with one study reporting that mental distress had doubled since the pandemic (Fisher et al., 2020).

The ensuing mental health crisis requires urgent, evidence-based, and interdisciplinary action (Campion et al., 2020; Holmes et al., 2020), and the use of digital technologies such as computers, tablets, and smartphones offers opportunities for the delivery of digital (i.e., Web- and app-based) interventions that are highly portable, accessible, and scalable. With primary care services urgently required to assist patients in clinical settings, evidence-informed digital mental health promotion interventions (DMHPIs) provide a pathway to enhance the mental health of the general population (Campion et al., 2020; Holmes et al., 2020; Marshall et al., 2020; Renfrew et al., 2020a; Przybylko et al., 2021a).

DMHPIs may improve the mental/emotional wellbeing of general population groups through a range of diverse approaches that enhance resilience and relieve stress, anxiety, and depressive symptoms. Suitable interventions may incorporate strategies from the fields of psychology (e.g., cognitive behavior therapy) (Lau et al., 2017; Cuijpers et al., 2019; Karyotaki et al., 2021), positive psychology (e.g., practicing gratitude, writing positive experiences, kindness) (Bolier et al., 2013; Drozd et al., 2014; Galante et al., 2014; Dickens, 2017; Hendriks et al., 2020; Koydemir et al., 2020), lifestyle medicine (e.g., physical activity, nutrition, access to green space) (Penedo and Dahn, 2005; Quirk et al., 2013; Cooney et al., 2014; Dale et al., 2014; Sarris et al., 2014, 2015; Cohen-Cline et al., 2015; Conner et al., 2015; Jacka et al., 2017) and various other practices which cross over several of the abovementioned fields (e.g., mindfulness, meditation) (Spijkerman et al., 2016; Galante et al., 2021).

Despite the advantages of digital delivery, high attrition (i.e., dropout) and low adherence (i.e., participants not engaging with the intervention as intended by designers) often compromise the outcomes of digitally-delivered interventions (Couper et al., 2010; Mohr et al., 2011, 2013b; Kelders et al., 2012; Richards and Richardson, 2012; Kohl et al., 2013; Short et al., 2014; Spijkerman et al., 2016; Linardon and Fuller-Tyszkiewicz, 2020). While there are numerous theoretical perspectives on factors that improve attrition and adherence (Ryan et al., 2018), a commonly purported facilitator is human support, often referred to as “guidance” (Mohr et al., 2011; Baumeister et al., 2014; Alkhaldi et al., 2016; Ebert et al., 2018; Ryan et al., 2018; Linardon and Fuller-Tyszkiewicz, 2020). It is important to evaluate the evidence regarding human support as a modifying factor in attrition and adherence to DMPHIs, as the addition of human support has cost implications (Mohr et al., 2013a; Morledge et al., 2013; Drozd et al., 2014; Michie et al., 2017), limits scalability, and requires the time of scarce human personnel (Mohr et al., 2013a). On the other hand, human support may improve adherence through prompting participants to engage, fostering participant accountability, strengthening motivation, and therefore optimizing outcomes (Mohr et al., 2011; Schueller et al., 2016).

This mini review evaluated the effectiveness of adding human support to DMHPIs for employees and general community groups. A search (using PubMed, reviews and other sources) revealed a paucity of randomized trials that directly compared supported with unsupported conditions for non-clinical groups in work and community settings. Hence, the search was widened to include suitable meta-analytic reviews to evaluate trends between guided and unguided conditions in the workplace and general community. This review also appraised the findings from recent studies conducted by the authors that compared different human support conditions amongst a general community cohort.

## Human Support

While early studies among clinical cohorts suggested that adding human support to digital interventions improved outcomes (Richards and Richardson, 2012; Baumeister et al., 2014), more recent studies have demonstrated mixed results (Shim et al., 2017). For example, some clinical studies have shown no difference in the outcomes of self-guided vs. supported digital interventions (Titov et al., 2015, 2016; Ivanova et al., 2016; Mira et al., 2017; Dear et al., 2018a,b; Eimontas et al., 2018; Zagorscak et al., 2018). Moreover, it is feasible that the requirement for added human support elements in DMHPIs for healthy non-clinical population groups may be different to unwell clinical population cohorts where symptoms of disease or disorder (e.g., general malaise, compromised motivation) may impede adherence (Mohr et al., 2013b; Deady et al., 2017; Karekla et al., 2019; Karyotaki et al., 2021).

### Workplace Settings

#### Meta-Analytical Reviews

Systematic reviews and meta-analyses, regarding the effectiveness of workplace DMHPIs, present mixed evidence with regards to the influence of the addition of human support (Stratton et al., 2017; Phillips et al., 2019). For example, in a systematic review and meta-analysis, by Phillips et al. (2019), guided interventions consistently demonstrated higher effect sizes than unguided in all the areas of measurement: stress (Hedges g = 0.76 for guided and g = 0.27 for unguided), depression (g = 0.48 and g = 0.23), anxiety (g = 0.48 and g = 0.26), burnout (g = 0.69 and g = 0.33), insomnia (g = 1.00 and g = 0.53), mindfulness (g = 0.57 and g = 0.36) and wellbeing (no guided studies, unguided g = 0.35). However, the authors recommended caution as heterogeneity was high in the pooled analysis of 22 RCTs and subgroup analyses were underpowered. Conversely, Stratton et al. (2017) demonstrated that supported conditions (g = 0.27) were similar in effects to unsupported (g = 0.22). However, different types of approaches revealed heterogenous effects in regard to human support. Cognitive behavior therapy demonstrated low effects in both conditions, stress management showed small effects for supported, but negative effects in unsupported conditions. Mindfulness, which was only administered in unsupported conditions, demonstrated moderate effects (Hedges g = 0.59).

Carolan et al. (2017) conducted a systematic review of 21 digital psychological interventions for employees and reported similar outcomes for both supported and unsupported interventions (guided g = 0.39, self-guided g = 0.34). Notably, in further examination of 7 studies that reported the highest adherence and lowest attrition, human support was only one of four common elements: human support (used in 5/7 studies); use of persuasive system design principles (all studies); 6- or 7-week program length (a mean of 6.6 week length compared to a mean of 8.1 weeks in the other 14 studies); and, the use of “secondary modalities” (e.g., emails, text messaging) in intervention delivery (6/7 studies).

#### Randomized Trials

Two randomized trials compared human support conditions in workplace settings. Allexandre et al. (2016), compared three support conditions (i.e., no support, group support, and group support plus therapist support) in a workplace digital mindfulness and stress management intervention. The addition of therapist support added no benefit, but general group support improved both outcomes and adherence compared to those who received no support. Albeit, overall, adherence was low−50% of participants never logged on to the intervention. Additionally, the intervention was digitally-delivered but support was provided face-to-face.

A pooled analysis of three RCTs (Zarski et al., 2016) compared human support in a digital stress management workplace intervention. Researchers identified that “content” support (i.e., personalized written feedback for tasks completed) and “adherence” support (i.e., monitoring module completion and sending reminders) did improve adherence behavior in comparison to only “administrative” support (i.e., provision of contact details for technical support). Although there were no significant differences in adherence between the “content” and “adherence” focused support, the time investment required by support personnel for the adherence-focused support was considerably lower, which highlights substantial cost implications dependent on different support variables (e.g., mode, intensity, and frequency).

### Community-Based Settings

#### Meta-Analytical Reviews

Meta-analytical reviews of studies in community settings generally demonstrated improved effects in supported conditions; although, authors of one meta-analytical review (Deady et al., 2017) refrained from evaluating differences between supported and unsupported conditions due to high heterogeneity. In a meta-analysis (Koydemir et al., 2020), which included 68 RCTs of positive psychology interventions for community cohorts, some delivered digitally and some delivered face-to-face, significant differences were found between self-guided interventions and those facilitated face-to-face. The effect size for the face-to-face condition (*d* = 0.32) was almost twice the self-guided condition (*d* = 0.17), although effect sizes were in the lower range for both conditions. A meta-analysis by Heber et al. (2017) evaluated the efficacy of Web- and computer-based studies for stress management amongst generally healthy adults. Sub-group analysis showed that supported and unsupported conditions demonstrated positive effects, though supported conditions (Cohen *d* = 0.64) were almost double the effect size of unsupported (*d* = 0.33).

#### Randomized Trials

While access to human support is often desired by non-clinical DMHPI users (Carolan and de Visser, 2018; Renfrew et al., 2020b), its effectiveness in improving adherence or outcomes is less convincing, based on three randomized studies that compared human support variables and targeted general community cohorts. In a three-pronged RCT (Morledge et al., 2013), participants from the general population were recruited to assess the effectiveness of a digital mindfulness intervention. Participants in one intervention group completed the activities with no added human support, while those in a second intervention group were encouraged to interact through an online message board that was hosted by a facilitator. Both groups demonstrated significant improvements compared to the control group; yet, no significant differences in measurement outcomes were observed between the two intervention groups. Notably, 85% of those in the message board group reported that the message board was of little or no value (Morledge et al., 2013). Mira et al. (2017), in an RCT designed to assist participants to cope with difficulties and depressive symptoms, compared an automated human support condition with the same condition plus weekly phone calls. Both groups improved significantly compared to control and no differences in attrition, adherence or outcomes were observed. Furthermore, participant satisfaction was high in both intervention groups.

In a digital relaxation training intervention (Alfonsson et al., 2016), self-selected participants from the general population were randomized to either “normal” treatment (i.e., black/white text files, weekly support from a trained therapist) or “enhanced” treatment (i.e., color text, video format, daily therapist support). Participants in both treatments improved significantly, irrespective of differing levels of support. The participant relationship with the support therapist did not predict adherence; rather, attrition, and non-adherence were predicted by low intrinsic motivation, low perceived treatment credibility, baseline stress symptoms, and focus on immediate behavior consequences rather than long term goals. Conversely, adherence was positively correlated with intrinsic motivation, education level, and treatment credibility, including belief in the effectiveness of the online format.

#### Recent Research by the Authors

We have previously reported the results of a 10-week DMHPI that was implemented in a general population setting. The interdisciplinary intervention, that integrated strategies from the fields of positive psychology and lifestyle medicine, compared the influence of three different modes of human support on outcomes (Renfrew et al., 2020a) and adherence (Renfrew et al., 2020b). Participants were randomized into groups that received differing levels of human support: automated email support only, automated email support with added personalized text messaging, and automated email support plus a weekly, facilitated, group videoconference session. Significant pre- to post-intervention improvements in outcomes were recorded in every group irrespective of the human support condition. Similarly, adherence (i.e., viewing weekly video content, and completing daily and weekly experiential activities) was not significantly different between the groups. Further, no differences in either adherence or improvements in wellbeing metrics were observed between those participants who were randomized to their first preference for human support and those who were not (Renfrew et al., 2020b). Nonetheless, despite the apparent lack of effect of human support, 45% of the entire study cohort indicated they would prefer to have *all* forms of human support available to them (Renfrew et al., 2020b). The reasons for this are unclear, it may be that participants like the autonomy to choose from a broad range of choices, even when they have no interest in accessing all options.

We reported low attendance at videoconference support sessions, with 36% of the participants allocated to this group never attending a session and only 18% attending seven or more of the 10 sessions (Renfrew et al., 2020b). Consequently, the human support experience for many participants in this group was effectually the same as for those in the automated emails only group. Nevertheless, secondary analysis revealed that participants who attended seven or more videoconference sessions did experience significantly greater improvements in overall mental health, vitality, depression and life satisfaction compared to those who attended less than seven sessions; although, numerous variables (e.g., motivation level) may have contributed to this difference (Renfrew et al., 2020a).

We also examined the influence of a DMHPI on the experience of “flourishing” (Przybylko et al., 2021b), which is a construct used to express a high level of human functioning. We found that the inclusion of text messaging support, or weekly videoconferencing support, had no additional benefit in the enhancement of flourishing. Notably, in our research we have observed highest attrition rates among participants who were randomized to a treatment group that involved the greatest level of human support. Participants in the aforementioned human support comparative study had agreed to take part regardless of which arm of the study they were allocated to, however, 32% of those assigned to the videoconferencing group—the highest level of human support offered—withdrew upon receiving notification of their allocation (Renfrew et al., 2020a). The dropout attrition was significantly greater (*P* = .009) than attrition in the other two arms of the study that involved mostly automated support. Furthermore, videoconferencing was the least desired form of support by participants—just 7% indicated it as their preferred support method. Interestingly, 22% of participants preferred email support, 26% preferred emails plus text messaging, and the remaining 45% desired access to all support forms.

To gain further insight into participant perspectives, we conducted a qualitative study (Renfrew et al., 2021) using inductive thematic analysis to examine user perceptions regarding facilitators of, and barriers to, adherence. The provision of human support did not feature as a perceived facilitator of adherence. Rather, perceived facilitators were engaging presentations, time availability, ease of accessibility, easy or interesting experiential activities, and personal motivation augmented by the perception that the intervention would be highly valuable. Overwhelmingly, the largest perceived barrier to adherence was lack of time, which is similar to that reported by other recent studies in workplace settings (Carolan and de Visser, 2018; Blankenhagel et al., 2019). Other barriers, some of them also time related, were presentation length, time consuming system design elements, technical issues, and a wide array of interpersonal factors that generally related to wellbeing (e.g., illness) or capacity (e.g., motivation). Notably, the addition of human support is not likely to assist in overcoming the abovementioned barriers, or further enhance the perceived facilitators of adherence.

The emerging, but limited body of evidence indicates that human support is not the panacea for attrition, adherence and outcomes in DMHPIs for a general community cohort; rather, the influences on adherence are multifactorial and complex. In a scoping review, Ryan et al. (2018) concluded that adherence to digital interventions is impacted by a range of technological, environmental and individual influences that require interdisciplinary cooperation (Ryan et al., 2018). Zarski et al. (2016) likewise concluded that many interindividual differences related to adherence remain unknown.

## Discussion

The evidence in this mini review indicates that DMHPIs may be implemented among work-based and general community cohorts without the need for added human support. While the meta-analytical reviews generally demonstrated that human support improved adherence and augmented positive effects, the few randomized trials indicated that self-guided conditions were comparable, in adherence, and outcomes, to conditions that included human support. Notwithstanding, the indication that general population cohorts often desire access to human support signifies that it is a desirable element that should be considered as an automated component in intervention design.

One possible option to incorporating automated human support, is a flexible, elective approach in which intervention creators provide participants with substantial autonomy to customize support along with other discretionary features (e.g., gamification). For instance, self-tailoring or personalizing an intervention might include choosing human support options from a range of automated modes (e.g., text message reminders, email notifications), and choosing the intensity and frequency of support, based on individual preference.

Identified in Self-determination Theory as one of three core basic needs for humans—*autonomy*, along with *relatedness* (i.e., connection) and *competence*—when fostered well, create the supportive conditions for intrinsic motivation to be optimized (Ryan and Deci, 2000), and greater intrinsic motivation has been linked to greater adherence (Mohr et al., 2011; Alfonsson et al., 2016). Importantly, the autonomy to choose from different automated support options, along with providing choice in other discretionary features may assist in stimulating intrinsic motivation to adhere to a program (Yardley et al., 2016), resulting in improved outcomes.

Finally, evidence that adding human support improves attrition, adherence, and outcomes of DMHPIs for general population groups is limited and mixed. Furthermore, it is evident that well designed, self-guided interventions can be effective in enhancing mental wellbeing. Nevertheless, human support is generally desired by intervention participants. Providing autonomy for participants to individualize or self-tailor an intervention, by selecting personal preferences for automated support and other discretionary features, might enhance participant experience and also bolster intrinsic motivation to adhere to a program—an area for further research. Carefully designed DMHPIs, that include automated forms of human support, should be urgently implemented in community settings to alleviate the COVID-19 pandemic-related decline in mental wellbeing.

## Author Contributions

MR and DM conceptualized the study and outlined the review structure. MR wrote the initial manuscript and all authors were involved in the revising and editing process.

## Conflict of Interest

The authors declare that the research was conducted in the absence of any commercial or financial relationships that could be construed as a potential conflict of interest.

## Publisher's Note

All claims expressed in this article are solely those of the authors and do not necessarily represent those of their affiliated organizations, or those of the publisher, the editors and the reviewers. Any product that may be evaluated in this article, or claim that may be made by its manufacturer, is not guaranteed or endorsed by the publisher.
